# A Rapid 3D Melanoma–Skin Organoid for High-Throughput Assessment of Tumor Dynamics and Drug Response

**DOI:** 10.3390/ijms27125314

**Published:** 2026-06-12

**Authors:** Gemma Nomdedeu-Sancho, Nicholas Edenhoffer, Anastasiya Gorkun-Roeder, Ola A. Gaser, Carlos Kengla, Allie Benton, David W. Mullins, Anthony Atala, Shay Soker

**Affiliations:** 1 Wake Forest Institute for Regenerative Medicine, Wake Forest University School of Medicine, Winston-Salem, NC 27101, USA; gemma.nomdedeusancho@wfusm.edu (G.N.-S.); nicholas.edenhoffer@wfusm.edu (N.E.); anastasiya.gorkun@advocatehealth.org (A.G.-R.); ola.gaser@wfusm.edu (O.A.G.); carlos.kengla@advocatehealth.org (C.K.); bentap21@wfu.edu (A.B.); 2 Department of Microbiology and Immunology, Wake Forest University School of Medicine, Winston-Salem, NC 27101, USA; david.mullins@wfusm.edu

**Keywords:** organoids, melanoma, in vitro models, drug testing

## Abstract

Melanoma is the most aggressive type of skin cancer, driven by early invasion, phenotypic plasticity, and frequent resistance to targeted therapies. Although genomic profiling informs treatment selection, genotype alone often fails to predict therapeutic response, underscoring the need for rapid and physiologically relevant functional testing platforms. Here, we present a three-dimensional melanoma–skin organoid (mSO) model that integrates primary skin cells with melanoma cell lines in a self-assembling, high-throughput format. The spherical mSOs recapitulate native human skin architecture, including a stratified epidermis and a dermal–hypodermal core, while supporting melanoma growth within an appropriate tissue microenvironment. In this niche, melanoma cells display epidermal spreading in radial growth-like patterns, outward invasion, and transcriptional shifts toward a pro-invasive phenotype. Using live confocal imaging coupled with a custom automated image analysis pipeline, we quantitatively measured tumor growth, migration beyond the organoid boundary, and interactions between melanoma cells and normal melanocytes. The mSOs also captured genotype-specific drug responses: *BRAF*-mutant melanoma cells were sensitive to BRAF and MEK inhibition, whereas *NRAS*-mutant, *BRAF*–wild-type cells were resistant to BRAF inhibition but remained responsive to MEK inhibition. Altogether, our mSO platform combines architectural and functional complexity with experimental scalability, providing a robust framework for modeling melanoma progression and evaluating targeted therapeutic responses within a relevant skin microenvironment. In the future, adaptation of this system to include patient-derived tumor cells could support personalized therapeutic decision-making in melanoma.

## 1. Introduction

Melanoma is the most lethal form of skin cancer because of its high invasive potential. Around 100,000 new cases of melanoma are diagnosed yearly in the United States [1], with incidence rates projected to continue rising in the coming years. Although only 4% of melanomas are metastatic at the time of diagnosis, metastasis is associated with a markedly reduced 5-year survival rate of approximately 27% [2,3]. The introduction of targeted therapies has significantly improved metastatic melanoma patient outcomes [4,5]. However, a significant proportion of patients either fail to respond to these therapies or develop resistance over time, ultimately leading to disease recurrence [6,7].

Melanoma is highly heterogeneous at both genetic and phenotypic levels. While genomic profiling has enabled patient stratification for targeted therapies, particularly in cases harboring activating mutations in the MAPK pathway, genotype alone is often insufficient to predict therapeutic durability. Tumor-intrinsic factors, including phenotypic plasticity, adaptive signal rewiring, and epigenetic state transitions, can contribute to therapeutic resistance, as can interactions with the tumor microenvironment (TME), the essential network of stromal cells, signaling molecules, and extracellular matrix that dictates invasion and drug sensitivity. Consequently, patients may be subjected to ineffective therapies that impose unnecessary toxicity and subsequently delay or undermine more effective treatments. Thus, there could be significant benefits from a high-throughput, fast-turnaround, and personalized drug-testing platform. Such a tool would provide clinicians with data to support the selection of the most effective therapy combinations, ultimately sparing patients from side effects or comorbidities associated with ineffective treatments. Current preclinical models have significant limitations in supporting this goal. Traditional two-dimensional melanoma cultures fail to predict in vivo human responses because they cannot recapitulate the complex interactions within the TME. Murine models provide a systemic context; however, fundamental physiological differences between mouse and human skin restrict their translational utility. Furthermore, patient-derived xenografts are time-consuming and costly, rendering them impractical for time-sensitive clinical decision-making.

Recent advances in melanoma spheroids, organoids, and skin equivalents have great potential to address these shortcomings. However, these models usually fail to reproduce the native layered architecture of the skin, a critical determinant of metastatic initiation [8,9]. Most existing models are either oversimplified, incorporating only two to three cell types, or too complex and resource-intensive for high-throughput screening [10,11,12,13]. As a result, there remains a critical unmet need for a platform that balances biological complexity with the rapid turnaround necessary for personalized medicine.

Our laboratory has recently developed a spherical, self-assembling human skin organoid model from primary skin cells. These organoids form a mature stratified epidermal layer containing keratinocytes and melanocytes surrounding a dermal–hypodermal core composed of fibroblasts and adipocytes [14]. In the present study, we extend this platform by including melanoma cell lines to create a high-throughput melanoma–skin organoid system optimized for rapid in vitro therapeutic screening.

## 2. Results

### 2.1. Generation of Four Cell-Type Skin Organoids (SOs) and Melanoma–Skin Organoids (mSOs)

**Figure 1:**
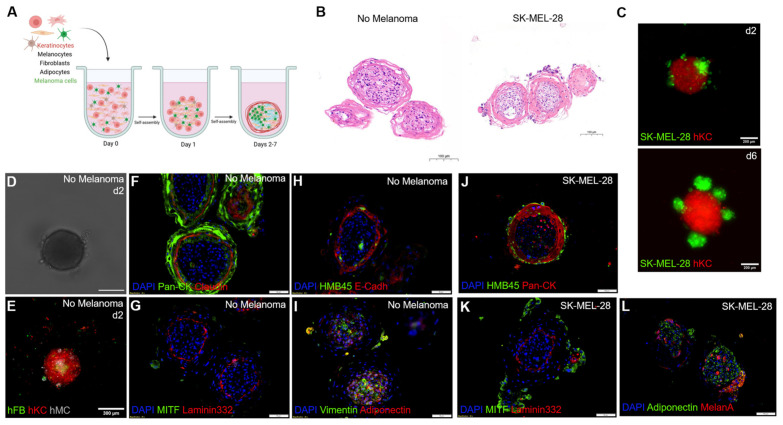
Generation of melanoma–skin organoids (mSOs) from primary skin cells and melanoma cell lines. (**A**) Workflow of mSO preparation. Created in Biorender. Gemma Nomdedeu-Sancho (2026) https://BioRender.com/. (**B**) H&E staining of paraffin-embedded skin organoids (SO) and mSOs containing SK-MEL-28 melanoma cells. (**C**) Live confocal images of mSOs on the second and last day of culture. Scale bars = 300 µm. (**D**) Bright field image of a SO on day 2 of culture. Scale bar = 300 µm. (**E**) Live confocal image of a SO on day 2 of culture. Scale bar = 300 µm. (**F**–**L**) Immunostainings at 20× magnification showing skin organoid cellularity and architecture, with and without melanoma cells. (hFB: human Fibroblasts, hKC: human keratinocytes, hMC: human melanocytes).

To generate four-cell-type skin organoids (SOs), we combined red fluorescent membrane-labeled human neonatal keratinocytes with primary human adipocytes, fibroblasts, and melanocytes. Cell ratios were adapted from our previous study [15] to maintain 10,000 cells per SO. The mixed cell suspension was seeded into 96-well ultra-low-attachment U-bottom plates, allowing spontaneous self-aggregation into spherical organoids, which were cultured for seven days. Melanoma–skin organoids (mSOs) were generated by adding GFP-labeled melanoma cells to the cell mixture (Figure 1A, Supplementary Figure S1). For this model, we selected the widely used melanoma cell line SK-MEL-28, which is derived from a metastatic cutaneous melanoma and harbors the *BRAF* V600E mutation [16]. By day 2 of culture, SOs formed compact spherical structures (Figure 1D) with the epidermal layer localized on the organoid surface, and fibroblasts confined to the core (Figure 1E).

Histological analysis of paraffin-embedded organoid sections demonstrated rapid epidermal maturation and stratification within 7 days of culture (Figure 1B, Supplementary Figure S2A). The epidermis exhibited keratinization and the presence of a Laminin-332^+^ basement membrane (Figure 1F,G, Supplementary Figure S2A). Interestingly, even without being exposed to an air–liquid interface (ALI), SOs displayed a similar epidermal maturation pattern to that observed in ALI-matured human skin equivalents (Supplementary Figure S2B). As expected, HMB45^+^ melanocytes were localized to the basal epidermal layer and were surrounded by E-cadherin^+^ keratinocytes (Figure 1H). The organoid core contained fibroblasts and adipocytes, resembling dermal and hypodermal compartments of the skin (Figure 1I). Collectively, these results demonstrate that the four-cell-type SOs rapidly self-organize into skin-like spherical constructs that preserve the skin’s appropriate cellular composition and layered architecture in vitro.

In skin organoids containing melanoma cells (mSOs), the addition of melanoma cells did not disrupt the SOs’ self-assembly, as structures remained round and compact. However, over time, melanoma cells proliferated extensively, invaded the epidermis, and migrated beyond the organoid boundary, forming nests on the epidermal surface (Figure 1B,C). Immunohistochemistry revealed melanoma invasion through the epidermis and outward migration, while overall SO integrity, cellularity, and architecture were preserved (Figure 1J–L). These features, combined with the system’s self-aggregating nature, establish mSOs as a robust and highly reproducible 3D platform for melanoma research. Following the initial assembly, the organoids require minimal maintenance beyond routine media changes every other day, reducing technical labor and experiment-associated variability.

### 2.2. mSOs Recapitulate the Early Stages of Melanoma Spreading and Invasion

In healthy skin, melanocyte proliferation and migration are tightly controlled through paracrine and juxtacrine signaling from adjacent keratinocytes [17,18,19]. During melanomagenesis, malignant melanocytes escape this control by secreting factors that downregulate Desmoglein1 (Dsg1) and E-cadherin expression in keratinocytes, facilitating horizontal spread within the epidermal layer before progressing to invasive stages, in a process known as the radial growth phase (RGP) [20,21].

**Figure 2:**
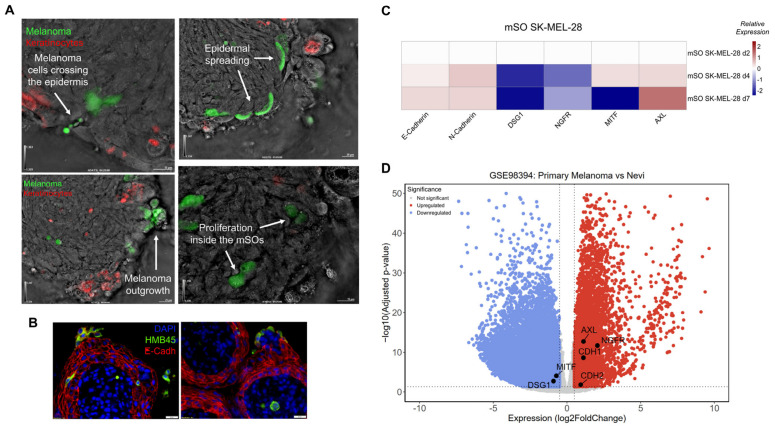
Melanoma cells invade the epidermal layer of the SOs, disrupt inter-keratinocyte junctions, and acquire a pro-invasive phenotypic signature. (**A**) Tomographic imaging of mSOs shows melanoma cells spreading and crossing the organoid epidermal layer to outgrow on the surface of the organoid, while some cells remain and proliferate inside. (**B**) Immunostaining of melanoma cells (HMB45^+^) crossing and budding off the epidermal layer (E-cadherin^+^). (**C**) qPCR analysis of SK-MEL-28 mSOs across three different timepoints indicates the disruption of inter-keratinocyte junctions and the acquisition of an invasive phenotype. Results show relative expression compared to day 2. (**D**) Volcano plot of significantly differentially expressed genes in human melanoma samples vs. benign nevi from the GSE98394 dataset. The expression pattern of pro-invasive genes in human samples matches the mSOs. Analysis using DESeq2.

To examine melanoma cell dynamics within the epidermal compartment, we performed live tomographic imaging of mSOs during early culture. GFP^+^ melanoma cells from the basal layer of the epidermis were observed migrating between keratinocytes toward the organoid surface (Figure 2A). A subset of cells remained within the epidermis, spreading radially in a pattern consistent with RGP (Figure 2A), whereas cells that reached and crossed the surface of the organoid proliferated and formed outward-growing tumor structures, indicative of a more migratory phenotype (Figure 2A). Immunostaining confirmed that HMB45^+^ melanoma cells crossed the E-cadherin^+^ epidermal layer and migrated outside the SOs (Figure 2B). A subset of melanoma cells, however, remained within the skin organoids and continued to proliferate (Figure 2A).

As melanoma progresses to invade the dermis in what is known as the vertical growth phase (VGP), tumor cells become more motile and migratory, undergoing a phenotypic switch from a proliferative, *MITF*^high^*/AXL*^low^, epithelial-like, and usually therapy-sensitive state to an invasive, *MITF*^low^*/AXL*^high^ state associated with undifferentiated, mesenchymal-like, and therapy-resistant phenotypes. Cells in different transcriptional states, including a neural crest cell-like state (NGFR^+^), may coexist within the same tumor, creating intratumoral heterogeneity in melanoma and driving tumor metastasis and treatment resistance.

Gene expression analyses of these melanoma progression markers revealed the downregulation of *DSG1* during early stages of melanoma dissemination, mimicking RGP, along with a progressive transition towards an *MITF*^low^*/AXL*^high^ transcriptional state over time (Figure 2C, Supplementary Figure S3), a hallmark of invasive melanoma [22,23,24]. The lack of *NGFR* expression indicated an invasive, dedifferentiated state in melanoma cells, but not a neural crest stem cell-like state. Together, these results demonstrate that mSOs recapitulate early melanoma formation, radial spreading, and the initiation of invasion within the skin’s structural environment.

Finally, to confirm the physiological relevance of the mSO system in recapitulating melanoma development in the skin, we cross-referenced our qPCR findings with publicly available bulk RNA-seq datasets from melanoma patients. We compared benign melanocytic lesions, representing a pre-melanoma stage equivalent to the first two days of mSO culture, against early- to late-stage primary melanomas. The clinical samples mirrored our in vitro data, showing significant downregulation of *DSG1* and *MITF* alongside increased expression of *AXL*, E-cadherin (*CDH1*), and N-cadherin (*CDH2*) (Figure 2D). These data validate our organoid system’s ability to capture in vivo pro-invasive transcriptional dynamics, establishing its suitability as a rapid and reliable human in vitro melanoma model.

### 2.3. Quantitative Analysis of Melanoma Growth, Migration, and Direct Interaction with Other Cell Types

**Figure 3:**
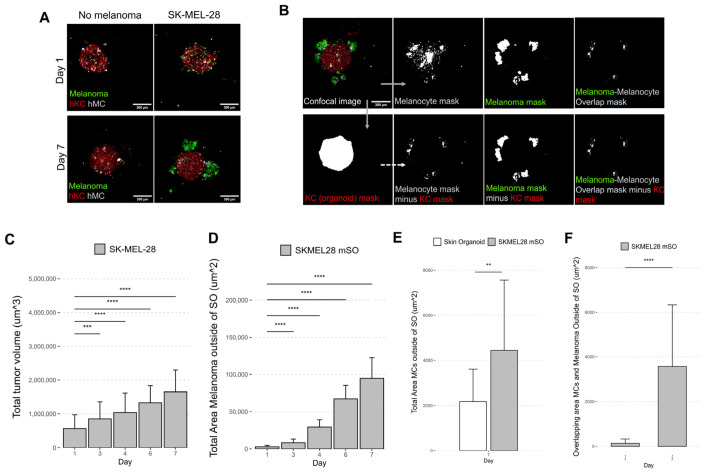
Quantification of tumor volume and budding of melanoma cells in the mSOs. (**A**) Live confocal images of SOs and mSOs with human melanocytes (hMC), human keratinocytes (hKC), and melanoma cells fluorescently labeled. (**B**) Workflow of the custom-made image analysis tool to calculate the area of melanoma, melanocytes, and overlapping melanoma–melanocytes outside of the skin organoids. (**C**) Quantification of total GFP^+^ melanoma cells (tumor volume) in mSOs. (**D**) Quantification of melanoma area outside of the mSOs. (**E**) Quantification of the total area of normal human melanocytes outside of the SO in the presence and absence of melanoma cells. (**F**) Quantification of the total overlapping area of melanoma and normal melanocytes outside the SO. *n* = 24 mSOs or SOs per condition. Statistical significance was assessed using repeated-measures one-way ANOVA with Holm-adjusted paired post hoc comparisons against day 1. Significance bars represent Holm-corrected paired *t*-tests following repeated-measures ANOVA. ** *p* < 0.01, *** *p* < 0.001, **** *p* < 0.0001. Dunnett’s post hoc test was used to validate the *t*-test results.

Given the heterogeneity of melanoma growth and migratory behavior across patient tumors, quantitative assessment of tumor growth and migration dynamics is essential for drug-screening applications. To quantify melanoma proliferation, we acquired live confocal images of the entire organoid and generated three-dimensional Z-stacks (Figure 3A). Total tumor burden was quantified by measuring the total GFP^+^ volume in each organoid using the 3D Objects Counter tool from Fiji-ImageJ (v.2.14.0/1.54p). We observed that SK-MEL-28 tumor volume increased significantly and consistently over time (Figure 3C), indicating sustained tumor growth within the organoids.

To quantify melanoma migration beyond the organoid boundary, we developed a custom image-analysis pipeline that measured the area of melanoma cells outside the SO. We generated maximum-intensity projections and used the red-fluorescent keratinocyte signal to create an organoid mask that defined the SO boundaries. The GFP^+^ melanoma signal was automatically thresholded, and the organoid mask was subtracted, enabling quantification of the tumor area outside the SO (Figure 3B, Supplementary Figure S1). Using this approach, we observed that melanoma expansion beyond the organoid occurred more rapidly than overall tumor volume growth (Figure 3D), consistent with a high migratory phenotype and aligned with the observed shift toward an *MITF*^low^/*AXL*^high^ state (Figure 2C). To compare melanoma cells exiting the organoids versus the cells remaining inside the organoids, we quantified the “out” vs. “in” melanoma area ratio. This ratio increased over time, confirming the progressive budding of melanoma cells (Supplementary Figure S4A) observed by tomographic imaging (Figure 2A). Proliferation occurred both inside and outside the organoid (Figure 2A), yet the area of melanoma inside the organoids remained constant (Supplementary Figure S4B). Assuming that the intrinsic proliferation rate of cell lines is expected to remain stable, a steady internal population paired with an expanding external population strongly suggests that this outward growth is driven by a combination of local proliferation with active migration from the interior. Consequently, this dynamic reflects the invasive behavior of melanoma within our 3D in vitro model.

Since mSOs also contain non-malignant human melanocytes, we investigated whether melanoma cells influence the behavior of normal melanocytes. mSOs were generated with Cy5 membrane-tagged melanocytes, which allowed simultaneous visualization of keratinocytes (red), melanoma cells (green), and melanocytes (far-red/gray in images). Confocal imaging revealed that normal melanocytes colocalized with SK-MEL-28 cells outside the organoid (Figure 3A), and quantitative spatial-overlap analysis confirmed a direct spatial association between migrating melanocytes and melanoma cells in this peripheral region (Figure 3F). Adapting the analysis pipeline described above, we further found that melanocyte exit beyond the SO border was significantly increased in the presence of melanoma cells compared with controls (Figure 3E). This correlation suggests that melanoma–melanocyte interactions occur at the invasive front and may be involved in melanocyte transformation. Our results demonstrate that the image analysis platform captures both melanoma growth, invasion, and complex, nuanced cell–cell interactions within the organoid microenvironment. In addition, this tool ensures robust and reproducible results through a semi-automated workflow: the ImageJ macros developed to process confocal images require no manual input or subjective adjustments, as a single fluorescence threshold is applied consistently to all images in the dataset for each fluorescent channel, ensuring consistent quantification within a set of images (Supplementary Figure S1).

### 2.4. mSOs Function as a High-Throughput Drug-Screening Platform

Different melanoma cell lines exhibit distinct growth and migration dynamics; therefore, they may respond differently to therapy. To evaluate the utility of mSOs and the associated image analysis pipeline for drug testing, we treated organoids with clinically used targeted therapies, including the BRAF inhibitor Encorafenib and the MEK inhibitor Trametinib. 5-FU was included as a non-specific chemotherapy. BRAF inhibitors (BRAFi), such as Encorafenib, selectively bind to the ATP-binding pocket of mutant BRAF V600E, suppressing constitutive MAPK activation resulting from the mutation. This results in the inhibition of tumor proliferation and the promotion of apoptosis [25]. On the other hand, MEK inhibitors (MEKi), such as Trametinib, act downstream of BRAF by binding to the catalytic sites of MEK1/2, thereby preventing ERK phosphorylation and activation, ultimately suppressing cell proliferation, while also inducing apoptosis [25]. Because BRAFi and MEKi target distinct nodes within the MAPK signaling pathway, they are commonly used in combination for the systemic treatment of patients with BRAF-mutant melanoma [26].

**Figure 4:**
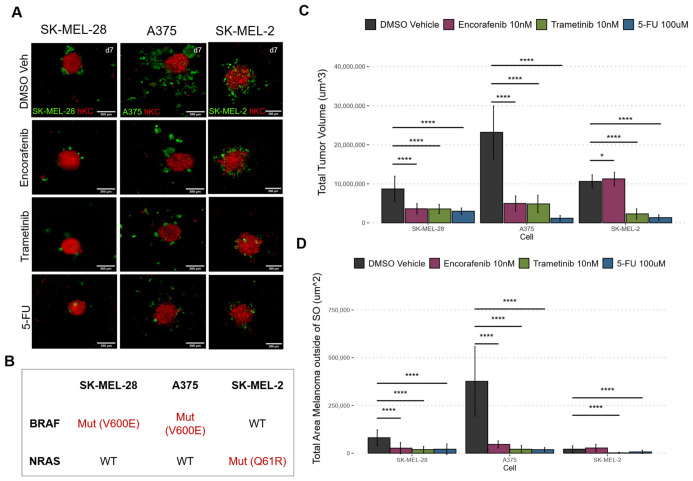
mSOs respond to targeted therapies and chemotherapies, reducing tumor volume and migration. (**A**) Live confocal images of mSOs treated with targeted therapies or 5-FU on the last day of culture. (**B**) Comparative table of the mutational status of the different melanoma cell lines. (**C**) Quantification of the tumor volume upon exposure of each treatment at the last timepoint. (**D**) Quantification of tumor area outside of the mSO upon exposure of each treatment at the last timepoint. *n* = 24 mSOs per condition. Two-way ANOVA revealed significant effects of treatment, cell line, and their interaction on tumor volume and area outside the SO. Significance bars show *t*-test results comparing the groups (different treatments) vs. control (DMSO Vehicle) within each cell line. All *p*-values are Holm-adjusted. * *p* < 0.05, **** *p* < 0.0001. Dunnett’s post hoc test was used to validate the *t*-test results.

To gain a broader view of the impact of the mutational background of melanomas on therapy outcomes, we generated mSOs with another melanoma cell line, A375, which also harbors the *BRAF* V600E mutation but is reported to exhibit a more aggressive phenotype than SK-MEL-28 [27]. A375 melanoma cells integrated efficiently into the mSOs and rapidly developed nests on the surface of mSOs, similar to those observed with SK-MEL-28 (Figure 4A). Because both SK-MEL-28 and A375 cell lines are *BRAF*-mutant, treatment with Encorafenib and Trametinib resulted in significant, time-dependent reductions in tumor volume and invasive area (Figure 4A,C,D). Notably, the therapeutic effects were more pronounced in A375 mSOs, consistent with their higher baseline proliferation and migration rates (Supplementary Figure S5). In contrast, SK-MEL-28 cells, which carry *TP53* and *PTEN* loss-of-function mutations alongside the *BRAF* V600E mutation [28], exhibited a more moderate response to treatment, consistent with reports showing that *PTEN* facilitates BRAFi resistance through the activation of the PI3K pathway [28,29,30]. These data highlight the sensitivity of our organoid platform in detecting nuanced treatment responses.

The doses of each inhibitor were selected based on IC50 curves for each melanoma cell line; however, to confirm that these effects reflected pathway-specific targeting rather than non-specific cytotoxicity, we generated mSOs using SK-MEL-2 cells, which are *BRAF* WT but harbor the *NRAS* Q61R mutation (Figure 4D). Although SK-MEL-2 cells integrated into the mSOs (Figure 4A) similarly to the *BRAF*-mutated cell lines SK-MEL-28 and A375, they were resistant to BRAF inhibition by Encorafenib but remained sensitive to MEK inhibition by Trametinib (Figure 4B,C). This selective response is consistent with the established therapeutic vulnerabilities of *NRAS*-driven melanomas, where mutated NRAS drives MAPK reactivation by promoting BRAF dimerization and subsequent downstream MEK activation [31]. Despite exhibiting growth and migration dynamics comparable to SK-MEL-28, SK-MEL-2 cells showed a distinct treatment response, underscoring the model’s ability to detect melanoma-intrinsic genetic dependencies (Figure 4).

Collectively, these results demonstrate that mSOs provide a robust, seven-day, high-throughput screening platform that integrates genetic drivers with physiologically relevant tumor microenvironment interactions to enable precise evaluation of melanoma behavior and therapeutic responses.

## 3. Discussion

The emergence of signal transduction inhibitors has revolutionized the treatment of melanoma [26,32]. However, durable responses remain limited, as many patients develop acquired resistance. In addition, while clinicians have access to multiple therapeutic agents targeting the same signaling pathways (for example, the BRAF inhibitors Vemurafenib and Encorafenib), these drugs frequently elicit different clinical responses in patients, likely due to the molecular heterogeneity of the melanoma. Consequently, therapeutic strategies effective in one *BRAF*-mutant melanoma patient may fail in another, underscoring the critical need for predictive platforms capable of distinguishing among these nuanced patient-specific responses.

While recently developed in vitro 3D melanoma models successfully capture features such as tumor plasticity [33,34], invasion [35], and drug response [34,36,37,38], they fail to integrate the crosstalk between these processes. This limitation likely stems from a lack of cellular diversity; most models incorporate only one or two stromal populations, failing to recapitulate the stratified skin architecture [10,14,39,40] that critically dictates melanoma progression and metastatic behavior. In contrast, planar skin equivalents are architecturally faithful; nonetheless, they are also labor-intensive and slow to generate, limiting their utility for high-throughput drug screening [11,13,41]. Consequently, current platforms require a tradeoff between microenvironmental complexity and scalability. Our model addresses these hurdles using a self-assembling primary skin cell-based organoid protocol that accurately reproduces native skin architecture and cellular composition while enabling rapid, scalable modeling of melanoma progression and therapeutic response.

In this study, we have demonstrated that mSOs accurately reproduce melanoma development and pathological behaviors, including radial growth in the epidermis and the initiation of invasion, in a fully human, structured microenvironment. A key strength of the mSO platform is its capacity to accept diverse melanoma cell lines while maintaining the skin architecture and cellularity of the skin organoids, mimicking the melanoma TME. Incorporation of *BRAF*-mutant melanoma cells with distinct proliferation and migration capabilities resulted in normal skin organoid architecture while enabling tumor growth in its native tissue context. For example, A375 cells exhibited greater proliferation relative to SK-MEL-28, consistent with their reported aggressive phenotype [27]. Importantly, this platform enabled accurate melanoma development in its native environment while maintaining the appropriate cell–cell interactions expected in the melanoma TME. Notably, we observed that SK-MEL-28 cells promoted the outward migration of normal melanocytes (Figure 3A,E,F), suggesting tumor-induced disruption of melanocyte homeostasis.

Furthermore, we developed an accompanying image analysis pipeline that enhances the platform’s utility by enabling automated, real-time quantitative assessment of tumor growth, invasion, and cell–cell interactions. By measuring overlapping, spatially resolved signals from different cell types within and outside the SOs, this automated workflow provides simultaneous readouts of tumor proliferation and invasive expansion, the latter serving as a proxy for the transition toward a pro-invasive phenotype. Because these quantifications are performed directly from live confocal images, our method bypasses the need for endpoint post-processing or fixation, even though these options remain available for further assessment of tumor characteristics. In addition, throughput can be high because organoids self-assemble, imaging can be automated, and image analysis does not require manual input. This rapid, scalable, and non-destructive readout is particularly advantageous for time-sensitive drug testing applications.

Preserving the intricate cell–cell interactions and broader TME features is essential during drug screening, as microenvironmental cues strongly influence drug sensitivity. Using our mSO platform, we accurately mimicked known genotype-specific treatment responses. As expected, *BRAF*-mutant cell lines (SK-MEL-28 and A375) exhibited sensitivity to Encorafenib, Trametinib, and 5-FU. Conversely, the *BRAF*-WT/*NRAS*-mutant line SK-MEL-2 was resistant to Encorafenib but responded to downstream MEK inhibition. This aligns with established literature demonstrating that single-agent BRAF inhibition paradoxically activates the MAPK pathway in *BRAF*-WT/*NRAS*-mutant melanomas [42,43]. Mechanistically, the inhibitor promotes BRAF-CRAF heterodimerization, which drives downstream MEK activation, enhances tumor proliferation, and mediates BRAFi resistance [31]. We have observed this pattern, as we quantified greater tumor volume in SK-MEL-2 mSOs treated with the BRAFi Encorafenib compared to controls (Figure 4B). These findings highlight the model’s ability to discern melanoma-intrinsic genetic dependencies within a complex tissue framework.

Although it was not explored in this study, preliminary data from our laboratory suggest that mSOs are suitable for evaluating the combination of targeted therapies with TME-directed agents such as FAK and ECM remodeling inhibitors, which have shown promise in the treatment of melanoma but have failed in clinical trials when used as monotherapies [44,45,46,47]. The mSO platform may offer a more physiologically relevant system to optimize these novel therapeutic combinations.

Beyond intrinsic genetics, our model also recapitulates the non-mutational transcriptomic reprogramming driving melanoma progression, mirroring human patient phenotypes through the transition toward an *MITF*^low^/*AXL*^high^ state (Figure 2C,D). Rather than relying strictly on clonal evolution, melanoma cells undergo a reversible phenotype switch to adapt to microenvironmental cues and expand intratumor heterogeneity [48]. Within the 3D mSO architecture, structural and cellular TME components may generate localized stressors that drive phenotype switching, including hypoxia, metabolic deprivation, and inflammation. These TME-derived stressors activate adaptive cascades, including the p38 MAPK, HIF1α, and the Integrated Stress Response (ISR) pathways, favoring the acquisition of an EMT-like state characterized by increased motility and drug tolerance [22,49]. Upon validation, the mSO platform could serve as a tool for dissecting how complex microenvironmental inputs promote transcriptomic reprogramming and acquired mechanisms of resistance.

Indeed, we observed that some tumor cells survive the targeted therapy, despite tumor volume being substantially reduced (Figure 4). Some of the remaining cells may already have developed resistance or be initially unresponsive clones. This observation is especially important in future experiments in which biopsy-derived tumor and stromal cells may be integrated to create patient-derived mSOs. These organoids could potentially provide a comprehensive therapeutic-sensitivity report and identify mechanisms of acquired resistance. Given the rapid timing for mSO production and analysis, this information could be delivered to clinicians promptly, facilitating personalized treatment decisions. Besides melanoma, the versatility of this system could be extended to other malignancies, including non-melanoma skin cancers and tumors with skin tropism, such as lymphomas. Modeling these tumors within a biomimetic skin niche will likely yield critical insights into their pathogenesis and uncover novel therapeutic targets.

Despite its strengths, the mSO model has important limitations. As a submerged system, it does not currently account for the effects of an air–liquid interface on tumor development, though we have demonstrated that epidermal maturation still occurs under these conditions. In addition, while the external positioning of the epidermis is optimal for therapy testing, it may not be the best approach for screening anti-metastatic therapies. Akin to what Van Kilsdonk and colleagues [9] described in their planar skin model, without an air–liquid interface, melanoma cells in our model migrate outward along the path of least physical resistance rather than traversing the dermal–epidermal junction (DEJ) [9]. Nonetheless, their adherence to the organoid and their phenotypic switch towards a pro-invasive transcriptomic *MITF*^low^/*AXL*^high^ state demonstrate that the microenvironment provides essential cues for survival and invasion. Currently, this model omits systemic factors (e.g., hormones) known to drive melanoma progression, as well as other critical TME cell types, such as immune and vascular cells. This limits its use for testing standard-of-care immunotherapies and anti-angiogenic drugs. While co-culturing mSOs with antigen-presenting cells (APCs), antigen-activated T cells, or skin-derived microvascular cells is possible, significant technical challenges remain in sourcing these cells and achieving their functional integration. Future iterations will focus on overcoming these hurdles, alongside protocol adaptations, to fully capture vertical growth phase (VGP) dynamics, systemic signaling, and advanced therapeutic responses.

In summary, we have developed a melanoma–skin organoid platform that combines architectural fidelity, biological complexity, and experimental scalability. By integrating the actions of specific genetic drivers with physiologically relevant TME interactions, this system provides a rapid, predictive framework that, when adapted to include patient-derived cells, could potentially inform personalized therapeutic strategies, advancing the goal of delivering the right treatment to the right patient at the right time.

## 4. Materials and Methods

### 4.1. Human Primary Cell Isolation

Primary keratinocytes, melanocytes, fibroblasts, and pre-adipocytes were isolated from discarded human foreskin samples with permission from the institutional IRB (protocol number 00007586). To minimize individual variation between samples, primary skin cells were isolated from more than 10 randomly selected patients under 1 year old and pooled from 3 or 4 donors for subsequent experiments. The same cell isolation procedure was performed on all samples. Briefly, the samples underwent three 10 min washes in Dulbecco’s Modified Eagle Medium (DMEM) low glucose (Hyclone; Logan, UT, USA) containing 10% Antibiotic/Antimycotic (AA) solution (Gibco; Grand Island, NY, USA) and 5% Amphotericin B (Gibco; Grand Island, NY, USA). The hypodermis was then separated from the dermis, minced into small pieces, and incubated in Type I collagenase (1 mg/mL) for 1 h at 37 °C. To stop the reaction, the minced hypodermis was filtered through a 70 µm mesh filter, placed in DMEM low glucose (Hyclone; Logan, UT, USA) with 10% fetal bovine serum (FBS) and 1% AA, and centrifuged at 1500 rpm for 5 min. After discarding the supernatant, the cells were washed again with the supplemented DMEM low glucose and seeded in tissue culture-treated 15 cm plates at a density of 8000 cells/cm^2^. The dermis and epidermis pieces were incubated overnight in 3–4 U/mL Dispase II (Worthington Biochemical Corporation; Lakewood, NJ, USA) diluted in DMEM low glucose basal medium (Hyclone; Logan, UT, USA) at 37 °C. The next day, the epidermis and dermis were separated using sterile forceps and collected separately. The epidermis was then digested in 5 mL of 0.25% trypsin/EDTA solution for 30 min at 37 °C, and the cells were released by pipetting with a 5 mL pipette. The trypsinized cells were then filtered through a 100 µm mesh filter and centrifuged at 1300 rpm for 5 min. The cell suspension was then split in half and plated in KGM2 medium for keratinocyte isolation and in MelM medium for melanocyte isolation (Gibco; Grand Island, NY, USA). To isolate the dermal fibroblasts, the dermis was minced using sterile curved scissors, resuspended, and seeded in DMEM High Glucose supplemented with 10% FBS and 1% AA and incubated at 37 °C for 2–3 days to allow fibroblasts to migrate from the explants onto the dish. Then, all cells were cultured as described below.

### 4.2. Cell Culture

SK-MEL-28-GFP^+^ melanoma cells were purchased from Angio-Proteomie (ref. Cap-0084GFP) and maintained in MelM medium supplemented with SupplementMix (Sciencell; Carlsbad, CA, USA) and passaged at 70–80% confluence. Melanocytes were also cultured in MelM medium and passaged at 80–90% confluence. A375 melanoma cells were purchased from ATCC (ref. CRL-1619), cultured in DMEM High Glucose medium supplemented with 10% FBS and 1%AA and passaged at 80–90% confluence. SK-MEL-2 cells were purchased from ATCC (ref. HTB-68) and cultured in DMEM Low Glucose medium supplemented with 10% FBS and 1% AA and passaged at 70–80% confluence. Primary keratinocytes were cultured in KGM2 (Promocell; Heidelberg, Germany) and passaged at 70% confluence. Isolated fibroblasts were cultured in DMEM High Glucose medium supplemented with 10% FBS and 1% AA and passaged at 90–100% confluence. Isolated pre-adipocytes were maintained in DMEM Low Glucose supplemented with 10% FBS and 1% AA and passaged at 90–100% confluence. To generate organoids, cells were used between passages 2–12.

### 4.3. Generation of GFP^+^ Melanoma Cell Lines

To generate the GFP^+^ A375 and SK-MEL-2 cell lines, cells at 70% confluence were transduced with the vector pLenti-CMV-GFP-Puro-LV (Addgene #17448) together with polybrene (Sigma Aldrich; St. Louis, MO, USA) (final concentration of 6 µg/mL) and maintained in DMEM supplemented with 10% FBS and 1% AA for two days. Transduced cells were then trypsinized and resuspended in fresh DMEM containing 2 µg/mL puromycin (ScienCell; Carlsbad, CA, USA). Puromycin selection was carried out for 1.5 weeks, with the medium being replaced twice a week. Selected cells were then expanded and frozen in stocks of 10 million cells.

### 4.4. Generation of Melanoma–Skin Organoids (mSOs)

To generate the melanoma–skin organoids (mSOs), cells growing in 2D were washed twice with DPBS, lifted using 0.05% trypsin (Gibco; Grand Island, NY, USA) solution, and centrifuged at 1200 rpm for 5 min. The resulting pellets were resuspended in their respective growth media (see Section 4.2). Cells were counted using the Countess3 cell counter (Thermo Fisher Scientific Inc.; Waltham, MA, USA). Keratinocytes were membrane-labeled using the Vybrant DiD fluorescent tag (Thermo Fisher Scientific Inc.; Waltham, MA, USA). Cells were then mixed in Mixed Media (MM) containing a 1:1:1:1:1:1 ratio of each cell-specific medium. The cell mixture contained 37% keratinocytes, 7% melanocytes, 22% fibroblasts, 32% adipocytes, and 2% melanoma cells. 100 µL of the cell mixture was placed in each of the wells of an Ultra-Low Attachment 96-Well Plate (Corning Costar; Kennebunk, ME, USA), which led to seeding 10,000 cells per well. The mSOs were incubated overnight at 37 °C to self-assemble. The next day, 100 µL of fresh MM was added to the plate. mSOs were maintained for 6–7 days, changing media every 2 days.

### 4.5. Generation of ALI-Exposed Planar Human Skin Equivalents (HSEs)

HSEs were kindly provided by Dr. McNutt’s laboratory at Wake Forest Institute for Regenerative Medicine (WFIRM). In brief, HSEs were generated using a sequential assembly approach incorporating the major dermal and epidermal cell types. The dermal compartment consisted of a fibrin hydrogel containing primary fibroblasts, preadipocytes, and HUVECs ((Thermo Fisher Scientific Inc.; Waltham, MA, USA, ref.C01510C) (0.5 × 10^5^ cells of each type) placed on Transwells. Keratinocytes (1 × 10^5^ cells) were seeded onto the dermal construct and expanded under submerged culture conditions for 8 days. HSEs were maintained in a 1:1:1:1 mixture of keratinocyte, adipocyte, fibroblast, and HUVEC media throughout the culture period. On day 8, cultures were transitioned to the air–liquid interface (ALI) and supplemented with 50 µg/mL ascorbic acid (Thermo Fisher Scientific Inc.; Waltham, MA, USA ref.J66601.06) to initiate epidermal differentiation and support barrier formation over the following 14 days. HSEs reached terminal differentiation between days 18–22 and were ready for downstream applications.

### 4.6. Drug Treatments

For targeted therapies and chemotherapies, mSOs were generated as previously described and treated one day after assembly with Encorafenib (SelleckChem; Houston, TX, USA) (final concentration of 10 nM), Trametinib (SelleckChem; Houston, TX, USA) (final concentration of 10 nM) or 5-FU (Sigma Aldrich; St. Louis, MO, USA #F6627-1G) (final concentration of 100 µM). Treatment doses were chosen based on their experimental IC50 values provided below (Table 1). 72 h after treatment, the medium was replaced with fresh, mixed medium containing diluted treatments at the same concentrations. mSOs were cultured for an additional 72 h, bringing the total culture time to 7 days.

IC50s were determined using CellTiter-Glo Luminiscent Cell Viability Assay (Promega; Madison, WI, USA) according to the manufacturer’s directions. Briefly, cells were seeded in 96-well plates at a concentration of 10,000 cells per well in 100 µL of medium and left to attach overnight. The next day, we added increasing concentrations of the distinct drugs from 0 up to 100,000 nM. 72 h after treatment, 100 µL of CellTiter-Glo reagent was incorporated, and the contents were mixed for 2 min in an orbital shaker to induce lysis. After stabilizing the plate and incubating at room temperature for 10 min, the luminescence signal was read in the SpectraMax M5 Microplate Reader (Molecular Devices; San Jose, CA, USA).

### 4.7. Proliferation Assay

To assess the proliferation rate of melanoma cells, 5000 melanoma cells (SK-MEL-28 or A375) were seeded in a 24-well tissue-treated plate in DMEM High Glucose supplemented with 10% FBS and 1% AA, and incubated overnight at 37 °C to allow attachment. The next day, the plate was introduced to the Incucyte^®^ Live-Cell Analysis System (Sartorius, Ann Arbor, MI, USA). Phase contrast images of the wells were acquired every 6 h for 48 h. The images were then analyzed using Incucyte’s AI Confluence program to extrapolate proliferation data. 

### 4.8. Scratch Wound Healing Assay 

40,000 melanoma cells were seeded in a 96-well tissue-treated plate in DMEM High Glucose containing 10% FBS and 1% AA and incubated overnight at 37 °C to allow attachment. SK-MEL-28 cells were seeded in MelM medium. Confluent cells were starved overnight by replacing the medium with DMEM High Glucose supplemented with 0.1% FBS and 1% AA. For SK-MEL-28, starvation medium consisted of MelM with 0.1% FBS, 100 U/mL penicillin, and 100 μg/mL streptomycin. The next day, the Incucyte Wound Maker tool (Sartorius, Ann Arbor, MI, USA) was used to scratch the wells. After two DPBS washes to remove detached cells and debris, new starvation medium was added to the wells. The plate was then placed in the Incucyte^®^ Live-Cell Analysis System (Sartorius, Ann Arbor, MI, USA). Each well was imaged every hour for 48 h using the 10× wide-mode objective. To analyze the stacked images (time-lapse), we used the Fiji-ImageJ (v.2.14.0/1.54p) macro *MRI_Wound_Healing_Tool.ijm* (Wound Healing Tool (RRID:SCR_025260) available at https://github.com/MontpellierRessourcesImagerie/imagej_macros_and_scripts/wiki/Wound-Healing-Tool (accessed on 8 September 2025) using the default settings. The resulting data were analyzed using a custom-made RStudio (v5.4.2) script that compares the wound area at all time points to calculate a percentage of wound closure, which is then compared among groups. 

### 4.9. Live Imaging and Image Processing

Live imaging of the spherical mSOs was performed using the ImageXpress Confocal HT.ai system (Molecular Devices, CA, USA) with a 10× objective. Images of the mSOs are shown as a maximum projection unless indicated otherwise.

### 4.10. Quantification of Melanoma Volume and Melanoma Outside of the mSO

ImageJ macros were developed to quantify melanoma volume and melanoma area outside of spherical mSOs. Confocal images of the spherical mSOs were saved as 3D Z-stacks. To calculate the volume of GFP^+^ signal (melanoma), the ImageJ plugin “3D Objects Counter” was used, which provided the volume in px^3^ of each GFP+ particle across the Z-stack. The graphs show the sum of GFP^+^ volume per organoid. To calculate the area of melanoma (GFP^+^/green) outside the skin organoid area (red labeled), confocal Z-stacks were projected into 2D images. The channels were split. In the red channel, the area of the largest particle was recorded as the area of the organoid, and a mask of that area was created. In the green channel, the GFP^+^ signal was thresholded. The organoid (keratinocyte) mask was applied and subtracted from the green particles, so only the area of GFP^+^ particles outside of the organoid area (red) was quantified. Graphs show the sum of melanoma (GFP^+^) area outside of the red-labeled organoid. The scripts for these ImageJ macros are publicly available at https://github.com/gnomdedeu/A-Rapid-3D-Melanoma-Skin-Organoid-for-High-Throughput-Assessment-of-Tumor-Dynamics-and-Drug-Response (accessed on 30 May 2026).

### 4.11. Tomographic Imaging

mSOs were collected 24 h after formation and transferred onto a custom-made slide containing chambers connected by channels to supply medium throughout the imaging time. The custom well slide had a #1.5H coverslip glass bottom. The slide was covered with a coverslip and placed in the TomoCube XT-X1 (Daejeon, Republic of Korea) outfitted with a Tokai Hit incubator stage to maintain temperature, humidity, and CO_2_ during time-lapse acquisition. Images were acquired in 140 μm z-stacks, capturing the bottom and edges of the mSOs. For each field, holotomographic and fluorescent image stacks were acquired with FITC and TRITC channels.

### 4.12. Histology and Brightfield Imaging

SOs and mSOs were fixed in a 4% paraformaldehyde (PFA) solution in DPBS overnight at 4 °C. Prior to paraffin processing, spherical SOs and mSOs were embedded in 2% agarose gels for easier manipulation and transferred to 70% ethanol. Organoids were paraffin-processed and molded. 5 μm sections were obtained using a microtome and placed on microscope slides, which were then stained with Hematoxylin and Eosin. Images were acquired with the 3DHistech Slide Scanner (Epredia; Kalamazoo, MI, USA) and processed with the Olympus OlyVIA software (v.4.1 Build 27564).

### 4.13. Immunohistochemistry and Fluorescence Imaging

A 2 h antigen retrieval treatment of 10 mM sodium citrate (pH = 6.0) was performed on paraffin sections. The tissue sections were then permeabilized with PBS-Triton-X (0.2%) for 15 min in a humidified chamber. Two 5 min TBST washes were followed by a 30 min incubation in protein-blocking solution (Dako; Carpinteria, CA, USA) at room temperature. Primary antibodies (Table 2) were diluted 1:100 in antibody diluent (Dako; Carpinteria, CA, USA) and incubated onto the sections overnight at 4 °C. After three 5 min TBST washes, the secondary antibodies Alexa Fluor 488 anti-mouse (Abcam, cat. no. ab150117) and Alexa Fluor 594 anti-rabbit (Abcam, cat. no. ab150088) were diluted 1:200 in antibody diluent (Dako; Carpinteria, CA, USA), placed onto the sections, and incubated for 1 h at room temperature. After three more 5 min TBST washes, the tissue sections were stained with DAPI and mounted with Prolong Gold without DAPI (Invitrogen; Eugene, OR, USA). The slides were imaged either with the Olympus BX63 fluorescence microscope and the Olympus cellSens Dimension software (v.4.3 Build 31056) or with the Olympus Slideview VS200 (Olympus) fluorescence slide scanner and the Olympus OlyVIA software (v.4.1 Build 27564).

### 4.14. RNA Extraction and Quantitative PCR

RNA was extracted from 12 to 16 mSOs per group and batch using the automated QiACube RNeasy micro kit (Qiagen; Germantown, MD, USA) according to manufacturer directions. Concentration and quality of the extracted RNA were measured using a NanoDrop spectrophotometer (ThermoFisher Scientific; Waltham, MA, USA). Samples exhibiting suboptimal RNA quality (A260/280 ratio < 1.7) were further purified using the RNA cleanup kit (Qiagen). cDNA synthesis was performed using the High-Capacity cDNA Reverse Transcription Kit (Applied Biosystems; Waltham, MA, USA). All the primers (Table 3) were designed using NCBI’s Primer-BLAST [50] and sourced from IDT (Integrated DNA Technologies, Inc.; Morrisville, NC, USA). Primers were validated by PCR amplification with random cDNA prior to use. qPCR analysis was performed using Power SYBR^®^ Green PCR Master Mix (Applied Biosystems; Waltham, MA, USA) on a QuantStudio 3 real-time PCR system (Applied Biosystems; Waltham, MA, USA). The program cycle was the following: Stage 1: Activation: 50 °C for 2 min; Stage 2: pre-soak: 95 °C for 10 min; Stage 3: Denaturation: 95 °C for 15 s; Annealing: 60 °C for 1 min; Stage 4: Melting curve: 95 °C for 15 s, 60 °C for 15 s, and 95 °C for 15 s. The qPCR was repeated twice, each containing three technical replicates per sample and gene. Raw data from the Quant Studio 3 were exported and analyzed in RStudio (v5.4.2), using the ΔΔCq method, which showed the expression of each gene as a fold increase or decrease relative to the housekeeping gene β-Actin and the control samples. To account for variability across biological replicates and experimental batches, relative gene expression was analyzed using a linear mixed-effects model with timepoint, gene, and their interaction included as fixed effects, and experimental batch as a random intercept. The significance of fixed effects was assessed using ANOVA on the fitted model. *p*-values from post hoc multiple comparisons were adjusted using the Holm method.

### 4.15. Analysis of Publicly Available Bulk RNA-Seq Datasets

The RNA-seq counts from the GSE98394 (https://www.ncbi.nlm.nih.gov/geo/query/acc.cgi?acc=GSE98394, accessed on 25 May 2026) dataset, deposited by Badal et al., [51] were downloaded from the Gene Expression Omnibus website. This dataset included transcriptomic information on benign melanocytic lesions (nevi) and treatment-naive primary cutaneous melanomas, ranging from early to late stage. RNA-seq counts and metadata for this gene set were imported into RStudio (v5.4.2), and rows with low gene counts (<10) were removed for subsequent analysis. Differential gene expression analysis was performed using the DESeq2 R package (v1.50.0) [52] with its default settings: raw read counts were normalized internally using the median-of-ratios method, which accounts for differences in sequencing depth and library composition. Gene-wise overdispersion was modeled using a negative binomial generalized linear model (GLM) and a parametric trend line. Hypothesis testing for differential gene expression between “common acquired nevus” and “primary melanoma” was carried out using the default Wald test. *p*-values were adjusted for multiple testing using the Benjamini–Hochberg false discovery rate (FDR) procedure. The alpha value of the results was set to 0.01. Genes with an adjusted *p*-value ≤ 0.05 and absolute log2 fold change ≥ 0.5 were considered significantly differentially expressed. To make the Volcano plot, we used the Enhanced Volcano package (v1.13.2) and mapped the position of the genes of interest onto it.

### 4.16. Statistical Analysis

All experiments were performed in triplicate unless stated otherwise. Quantitative results are presented as mean ± standard deviation. The significance of data comparisons was evaluated using a two-way ANOVA for multiple group comparisons and a two-tailed Student’s *t*-test for pairwise comparisons. For qPCR analysis, a linear mixed-effects model was used. All *p*-values are Holm-adjusted. Significance values and details of the statistical analysis are indicated in the figure legends.

## Figures and Tables

**Table 1:** IC50s of drug treatments in the different melanoma cell lines.

	SK-MEL-28	A375	SK-MEL-2
Encorafenib	0.11 nM	1.7 nM	9934 nM
Trametinib	0.21 nM	0.14 nM	8.06 nM
5-FU	88.78 µM	11.32 µM	174.2 µM

**Table 2:** List of antibodies used for IHC experiments.

1º Antibody	Reference
Pan-cytokeratin	#PA1-27114 or MA5-13203. (Invitrogen; Eugene, OR, USA)
Keratin 14 (K14)	#PA5-32460 (Abcam; Waltham, MA, USA)
E-Cadherin	#ab40772 (Abcam; Waltham, MA, USA)
Adiponectin	#MA1-054 or #ab231100 (Invitrogen; Eugene, OR, USA) (Abcam; Waltham, MA, USA)
Vimentin	#ab8978 or #MA5-16409 (Abcam; Waltham, MA, USA) (Invitrogen; Eugene, OR, USA)
HMB45	#M0634 (Dako; Carpinteria, CA, USA)
SOX10	#ab180862 (Abcam; Waltham, MA, USA)
MelanA	#ab210546 (Abcam; Waltham, MA, USA)
MITF	#NB100-56561 (NovusBio: Centennial, CO, USA)
Claudin	#ab307692 (Abcam; Waltham, MA, USA)
Laminin-332	#711306 (Invitrogen; Eugene, OR, USA)
Involucrin	#29665 CellSignaling Technology: Danvers, MA, USA.
Keratin 10 (K10)	#ab76318 (Abcam; Waltham, MA, USA)
Connexin 43 (CNX43)	#13-8300 (Invitrogen; Eugene, OR, USA)

**Table 3:** List of primers used for qPCR experiments.

Gene	FW Primer	RV Primer	Size (bp)
E-Cadherin	GTGGTTCAAGCTGCTGACCTTC	ACCTGACCCTTGTACGTGGTG	122
N-Cadherin	GCGTCTGTAGAGGCTTCTGG	TGCAGTTGCTAAACTTCACATTGAG	130
Dsg1	ACTGACGCAGATGAACCGAAC	ACTGGCCGTATTGCTCTCTGTC	154
MITF	GCAGTGGAAGGACGGGAAG	TCGGCGGAACTGCTGC	143
AXL	GCTGGGAGCCCAACAACTTC	GCCTGCGTGCCCCTG	134
NGFR	ACATAGCCTTCAAGAGGTGGAAC	CCACGGAGATGCCACTGTC	123

## Data Availability

The raw data supporting the conclusions of this article will be made available by the authors on request.

## Supplementary Materials

The following supporting information can be downloaded at: https://www.mdpi.com/article/10.3390/ijms27125314/s1.
