# Supplementary material for: A Rapid 3D Melanoma–Skin Organoid for High-Throughput Assessment of Tumor Dynamics and Drug Response
Source: Int J Mol Sci. 2026 Jun 12;27(12):5314. doi: 10.3390/ijms27125314 (PMC13300543; doi:10.3390/ijms27125314)
Supplement: Supplementary file 1 [file ijms-27-05314-s001.zip › Figures.pdf]

SUPPLEMENTARY DATA

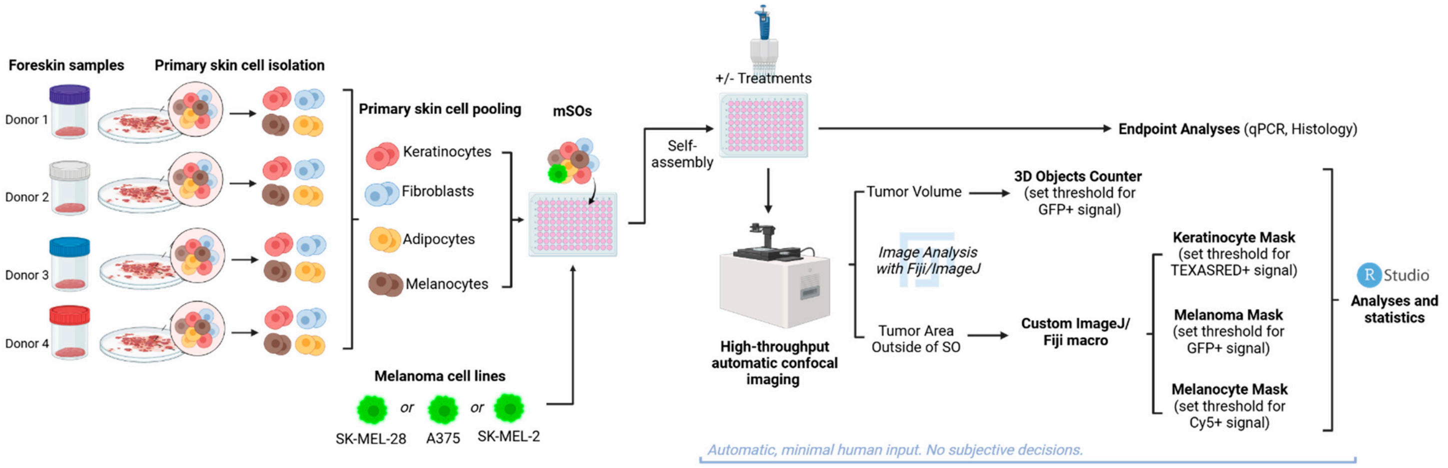

Supplementary Figure S1. Experimental workflow. Primary skin cells were isolated from foreskin samples from different donors and pooled together to generate mSO containing melanoma cell lines. After self-assembly, mSOs were cultured for 7 days. For some experiments, mSOs were treated with targeted therapies and chemotherapies. To quantify tumor volume and migration, mSOs were imaged using an automatic confocal microscope. The confocal images were processed in ImageJ/Fiji using consistent fluorescence thresholds across all datasets, thereby minimizing internal variability. The image analysis required only that users provide the image sets and import the results into RStudio for subsequent analyses and statistics. *Created with BioRender.*

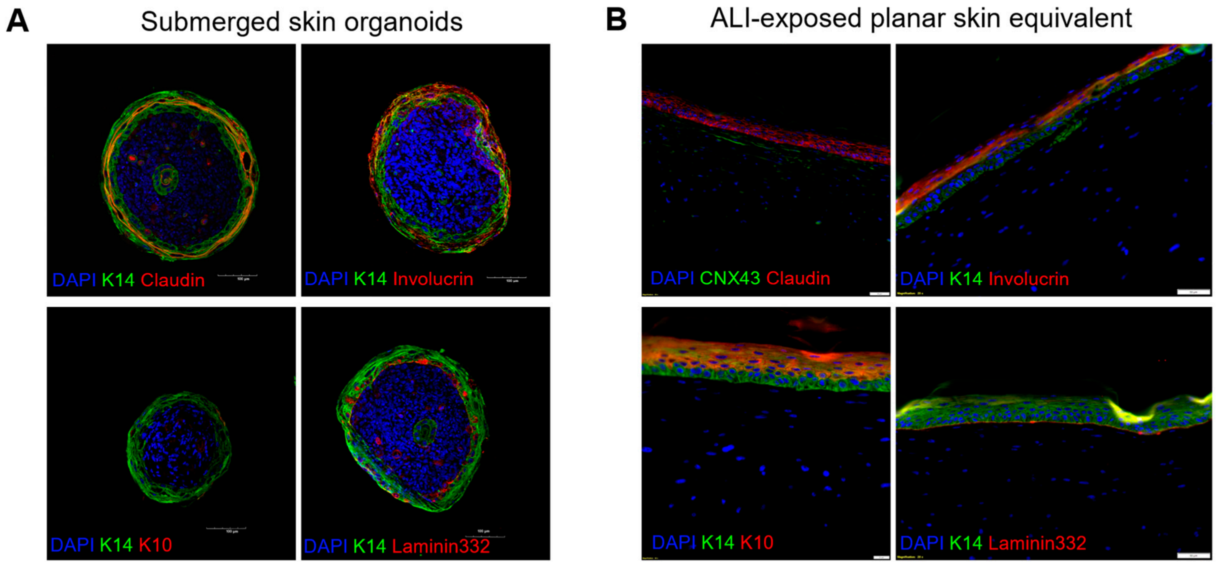

Supplementary Figure S2. The epidermal layer of submerged skin organoids (SOs) matures akin to air-liquid-interphase (ALI)-exposed planar skin equivalents. A. Immunostainings of epidermal maturation in 7-day-old skin organoids. **B**. Immunostainings of epidermal maturation in 14-day-old ALI-exposed planar human skin equivalents.

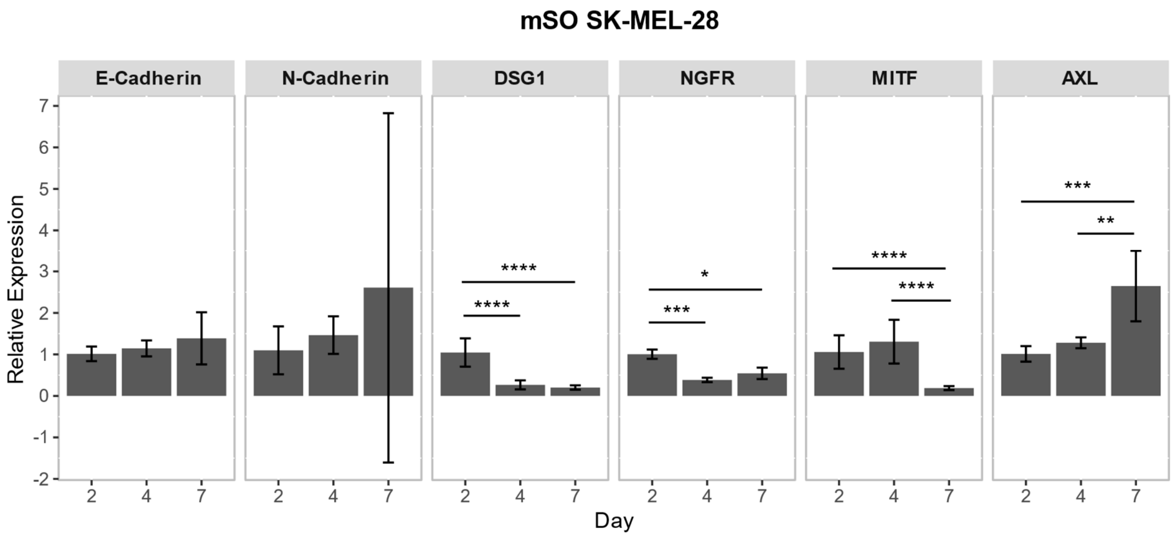

Supplementary Figure S3. Gene expression analysis in mSOs generated with SK-MEL-28 cells. Relative gene expression of melanoma invasion and progression-related genes across time in SK-MEL-28 mSOs. N=2 experiments, n=3 technical replicates per experiment. Data represent 2^-(ΔΔCq) values. Relative gene expression was analyzed using a linear mixed-effects model with timepoint, gene, and their interaction as fixed effects and experimental batch as a random intercept. *P-values were Holm-adjusted. *p<0.05, **p<0.01, ***p<0.001, ****p<0.0001.*

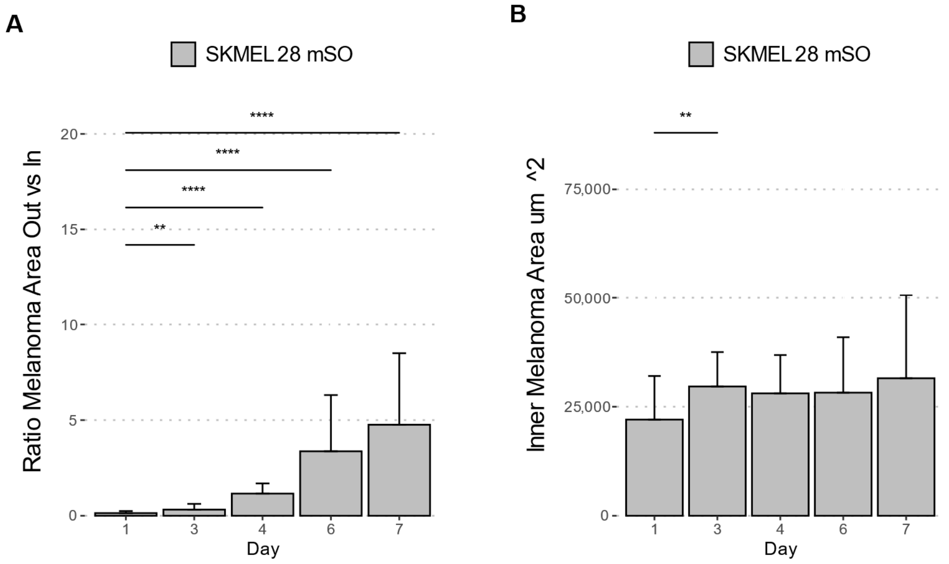

Supplementary Figure S4. Melanoma invasion dynamics in the mSOs. **A.** Quantification of the melanoma area ratio outside vs. inside the SOs across the organoid culture period. **B.** Quantification of area of melanoma cells inside the skin organoid throughout culture time. *n = 24 mSOs per condition. Statistical significance was assessed using repeated-measures one-way ANOVA with Holm-adjusted paired post-hoc comparisons against day 1. Significance bars represent Holm-corrected paired T-tests following repeated measures ANOVA. *p<0.05, **p<0.01, ***p<0.001, ****p<0.0001.*

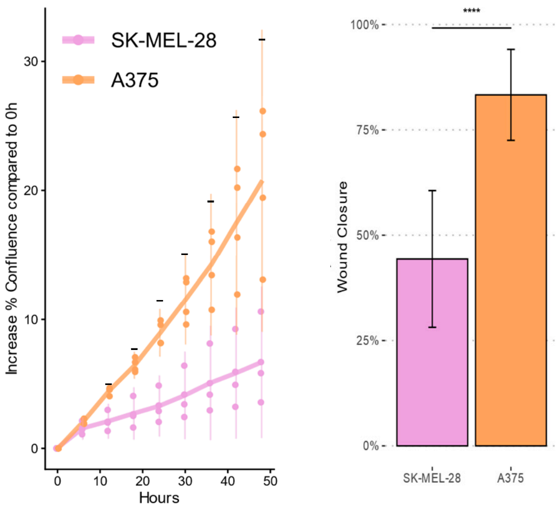

**Supplementary Figure S5. Melanoma cell proliferation and migration in 2D cell cultures**. **A.** Increase in cell confluence of SK-MEL-28 and A375. Results are shown as % increase relative to the initial seeding day. **B.** Wound closure at 48h from a scratch wound migration assay of SK-MEL-28 and A375. Results are shown as % of surface coverage. *T-Test *p<0.05, **p<0.01, ***p<0.001, ****p<0.0001.*
